# Supplementary material for: Unraveling TNXB Epigenetic Alterations Through Genome-Wide DNA Methylation Analysis and Their Implications for Colorectal Cancer
Source: Int J Mol Sci. 2025 Jul 25;26(15):7197. doi: 10.3390/ijms26157197 (PMC12346618; doi:10.3390/ijms26157197)

Supplementary Figure S1. Characterization of tumor-related colorectal cancer DMCpGs. A) Supervised clustering heatmap of the 5,000 DMCpGs that were found to be differentially methylated between the tumor and the NAT area. B) Filtered analysis to select genes thar are largely methylated and most likely to act as an epimutation

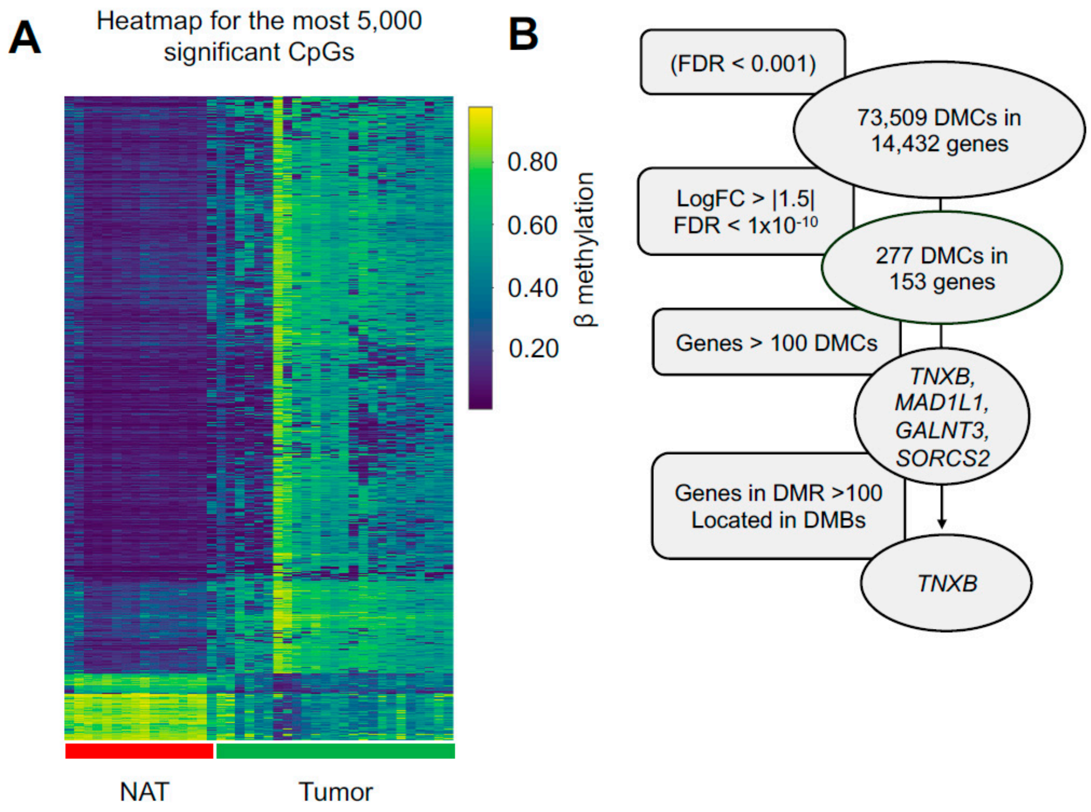

Supplement: Supplementary file 1 [file ijms-26-07197-s001.zip › Supplementary Figure S1.pdf]
