# Supplementary material for: Unraveling TNXB Epigenetic Alterations Through Genome-Wide DNA Methylation Analysis and Their Implications for Colorectal Cancer
Source: Int J Mol Sci. 2025 Jul 25;26(15):7197. doi: 10.3390/ijms26157197 (PMC12346618; doi:10.3390/ijms26157197)

**Supplementary Figure S2.** A) Single cell RNA-Seq analysis extracted from: https://www.ebi.ac.uk/gxa/sc/experiments/E-MTAB-8410/results/tsne?geneId=ENSG00000168477&plotOption=20&plotType=umap&colourBy=inferred+cell+type+-+authors+labels&markerGeneK=13&k=13 and from Lee HO et al (2020). B) Representation of DMR1 and DMR5 of the *TNXB* gene, and their three islands contained in the DMR 1. C) Cox regression analysis adjusted by cofounding variables. D) Kaplan–Meier analysis was performed to determine overall survival according *TNXB* gene methylation (low vs. high) from our study using the median of the *TNXB* methylation, and from the TCGA-COAD. E) ROC curve and cut-off for the predictive value of survival rate of *TNXB* methylation.

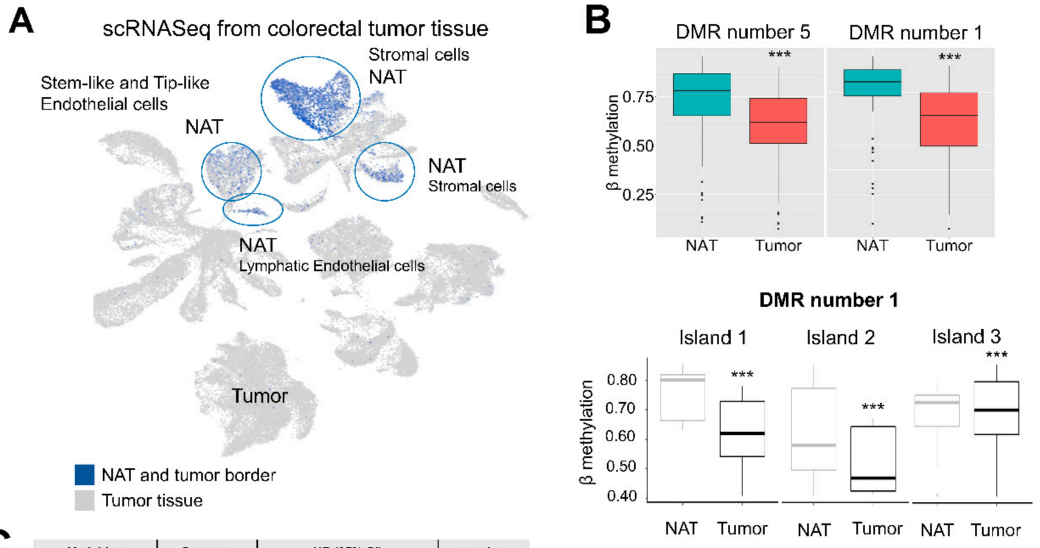


C

| Variables | Compare | HR (95% CI) | *p* value |
|---|---|---|---|
| *Unadjusted* | | | |
| *TNXB* methylation | T1+T2 vs T3 | 3.50 (1.00 – 12.25) | 0.049* |
| *Adjusted by age and localization* | | | |
| *TNXB* methylation | T1+T2 vs T3 | 6.74 (1.01 – 44.80) | 0.048* |
| *Adjusted by age, sex and localization* | | | |
| *TNXB* methylation | T1+T2 vs T3 | 9.26 (0.82 – 104.83) | 0.072 |

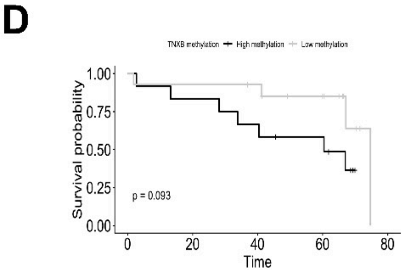


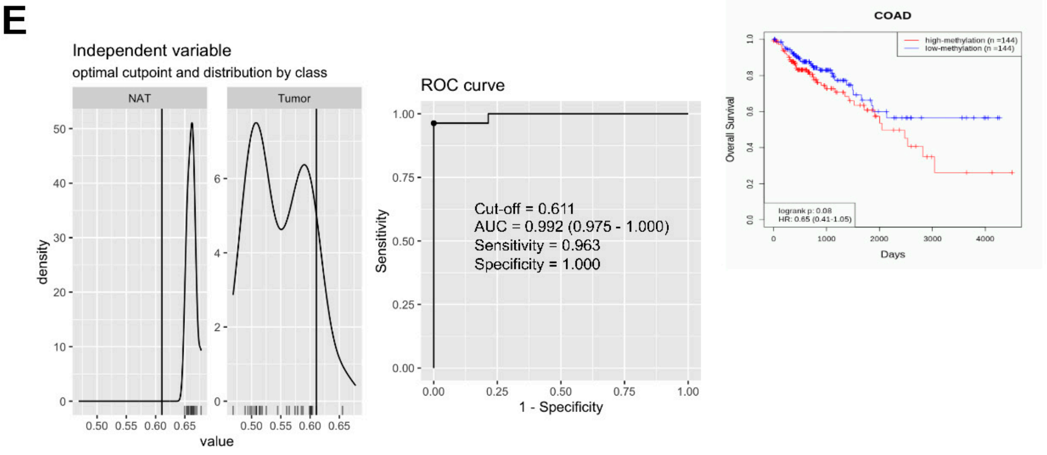

Supplement: Supplementary file 1 [file ijms-26-07197-s001.zip › Supplementary Figure S2.pdf]
