# Supplementary material for: Unraveling TNXB Epigenetic Alterations Through Genome-Wide DNA Methylation Analysis and Their Implications for Colorectal Cancer
Source: Int J Mol Sci. 2025 Jul 25;26(15):7197. doi: 10.3390/ijms26157197 (PMC12346618; doi:10.3390/ijms26157197)
Supplement: Supplementary file 1 [file ijms-26-07197-s001.zip › Supplementary Table S1.pdf]

Table S1. Baseline characteristics of patients with colorectal cancer included in the epigenome-wide DNA methylation assay.

Variables	All<br>*N=28*
**Demographic variables**	
Age (years)	65.8 (9.67)
Sex	
Male	19 (67.9%)
Female	9 (32.1%)
BMI (kg/m^2)	26.8 (4.07)
Lean (< 25 kg/m^2)	10 (35.7%)
Overweight (between 25 and 30 kg/m^2)	13 (46.4%)
Obese (≥30 kg/m^2)	5 (17.9%)
**Biochemical variables**	
Glucose (mg/dL)	126 (51.2)
Triglycerides (mg/dL)	173 (98.9)
Total cholesterol (mg/dL)	164 (36.6)
HDL (mg/dL)	37.2 (11.1)
LDL (mg/dL)	100 (29.0)
CRP (mg/L)	12.8 (16.9)
Hba1c (%)	5.97 (1.10)
HOMAIR	1.56 (1.53)
25-hydroxyvitamin D (ng/mL)	30.2 (15.1)
CEA (mg/dL)	3.92 (4.32)
CA19.9 (U/mL)	17.6 (19.3)
**Global methylation in tumor tissue (%)**	
Global methylation in tumor (*LINE1*)	54.3 (5.02)
Global methylation in NAT (*LINE1*)	62.6 (2.47)*
**Clinicopathological variables**	
Tumor location	
Right-sided colon	4 (14.3%)
Left-sided colon	7 (25.0%)
Rectum	17 (60.7%)
Cancer stage	
Stage I	11 (39.3%)
Stage II	7 (25.0%)
Stage III	8 (28.6%)
Stage IV	2 (7.14%)
T:	
2	13 (46.4%)
3	10 (35.7%)
4	3 (10.7%)
X	2 (7.14%)
N:	

Variables	All *N=28*
0	18 (64.3%)
1	6 (21.4%)
2	2 (7.14%)
X	2 (7.14%)
M:	0.07 (0.26)
No	26 (92.8%)
Yes	2 (7.20%)
Survival	
Yes	17 (60.7%)
No	11 (39.3%)

Data are presented as mean (standard deviations) or number of frequencies. Asterisk indicates significant difference between global DNA methylation measured as *LINE1* methylation in the tumor area in comparison with the NAT area, according to Wilcoxon paired-test (*p<0.05). **Abbreviations**: 25 (OH)D: 25-hydroxyvitamin D; BMI: body mass index; CA19.9: cancer antigen type 19.9; CEA: Carcinoembryonic antigen; CRP, C-reactive protein; Hba1c: Glycosylated hemoglobin; HOMA-IR: homeostasis model assessment of insulin resistance; HDL: high density lipoprotein cholesterol; NAT: Normal adjacent-tumor; *LINE1*: long-interspersed nucleotide element 1; LDL: low density lipoprotein cholesterol.
