# Supplementary material for: Unraveling TNXB Epigenetic Alterations Through Genome-Wide DNA Methylation Analysis and Their Implications for Colorectal Cancer
Source: Int J Mol Sci. 2025 Jul 25;26(15):7197. doi: 10.3390/ijms26157197 (PMC12346618; doi:10.3390/ijms26157197)
Supplement: Supplementary file 1 [file ijms-26-07197-s001.zip › Supplementary Table S3.pdf]

Supplementary Table S3. Top of the most 20 significant differentially methylated CpGs.

CpG name	Chromosome	Relation to island	Gene name	Relation to gene	Fold change	*p* value	FDR
cg08096684	4	Open Sea	*NA*	NA	-2,06	$9{,}18x10^{-23}$	$2{,}05x10^{-17}$
cg17698295	8	Island	*OPLAH*	DMR	3,41	$1{,}10x10^{-22}$	$2{,}05x10^{-17}$
cg17301223	8	Island	*OPLAH*	DMR	3,14	$1{,}35x10^{-22}$	$2{,}05x10^{-17}$
cg09087503	7	Island	*SND1*	DMR	3,31	$5{,}21x10^{-22}$	$5{,}94x10^{-17}$
cg21995919	2	Island	*ITGA4*	DMR	3,21	$3{,}31\ 10^{-21}$	$3{,}02x10^{-16}$
cg06193628	2	Open Sea	*NA*	NA	2,17	$5{,}35x10^{-21}$	$4{,}07x10^{-16}$
cg11258943	9	Island	*FOXE1*	NA	3,27	$8{,}88x10^{-21}$	$5{,}79x10^{-16}$
cg27200446	6	Island	*MDFI*	DMR	3,28	$1{,}28x10^{-20}$	$7{,}34x10^{-16}$
cg14012294	5	Island	*BTF3*	NA	-1,59	$7{,}56x10^{-20}$	$3{,}83x10^{-16}$
cg06952671	2	Island	*ITGA4*	DMR	3,14	$9{,}28x10^{-20}$	$4{,}23x10^{-15}$
cg17477445	12	Island	*DPY19L2*	DMR	2,75	$1{,}11x10^{-19}$	$4{,}63x10^{-15}$
cg02970297	7	Island	*DPY19L2P*	NA	2,17	$1{,}42x10^{-19}$	$5{,}42x10^{-15}$
cg17494199	13	Open Sea	*NA*	NA	-1,95	$2{,}00x10^{-19}$	$6{,}60x10^{-15}$
cg05649391	11	North Shore	*MYBPC3*	NA	-2,66	$2{,}02x10^{-19}$	$6{,}60x10^{-15}$
cg19932901	10	Open Sea	*NA*	NA	1,24	$2{,}85x10^{-19}$	$8{,}69x10^{-15}$
cg06706670	1	Open Sea	*UBR4*	NA	1,75	$4{,}89x10^{-19}$	$1{,}39x10^{-14}$
cg07080358	2	Island	*CNRIP1*	NA	2,62	$6{,}01x10^{-19}$	$1{,}61x10^{-14}$
cg15336765	12	Island	*AQP5*	DMR	3,15	$7{,}54x10^{-19}$	$1{,}91x10^{-14}$
cg16300300	13	North Shore	*NA*	cDMR	2,85	$9{,}63x10^{-19}$	$2{,}31x10^{-14}$
cg02001991	4	Open Sea	*NA*	NA	-1,70	$1{,}05x10^{-18}$	$2{,}40x10^{-14}$

Abbreviations: AQP5: Aquaporin 5; BTF3: Basic Transcription Factor 3; cDMR: cancer differentially methylated region; CNR1P1: Cannabinoid Receptor 1; DMR: differentially methylated region; DPY19L2: Dpy-19 Like 2; FDR, false discovery rate, FOXE1: Forkhead Box E1; ITGA4: Integrin Subunit Alpha 4; MDFI: MyoD Family Inhibitor; MYBPC3: myosin binding protein C3; NA: not available; OPLAH: 5-Oxoprolinase, ATP-Hydrolysing; SND1: Staphylococcal Nuclease And Tudor Domain Containing 1; UBR4: Ubiquitin Protein Ligase E3 Component N-Recognin 4.
