# Supplementary material for: Unraveling TNXB Epigenetic Alterations Through Genome-Wide DNA Methylation Analysis and Their Implications for Colorectal Cancer
Source: Int J Mol Sci. 2025 Jul 25;26(15):7197. doi: 10.3390/ijms26157197 (PMC12346618; doi:10.3390/ijms26157197)
Supplement: Supplementary file 1 [file ijms-26-07197-s001.zip › Supplementary Table S4.pdf]

Supplementary Table S4. Top of the most 20 significant differentially methylated regions.

DMR	Chromosomes	Start	End	Harmonized FDR	Number of CpGs	Overlapping genes
DMR1	6	32034322	32059605	$4{,}57x10^{-7}$	231	*TNXB**
DMR2	6	33128825	33151274	$9{,}12x10^{-7}$	171	*COL11A2*
DMR3	6	29520527	29521803	$1{,}85x10^{-7}$	40	*OR2I1P*
DMR4	7	27140797	27150598	$1{,}76x10^{-7}$	55	*HOXA-AS2*
DMR5	6	32014476	32033307	$4{,}74x10^{-7}$	157	*TNXB**
DMR6	13	78491982	78494462	$1{,}97x10^{-8}$	49	*RNF219-AS1*
DMR7	6	32141257	32159933	$6{,}74x10^{-8}$	142	*RNF5*
DMR8	1	119526060	119532925	$1{,}2x10^{-7}$	46	*TBX15*
DMR9	3	62353312	62365402	$6{,}2x10^{-8}$	63	*PTPRG-AS1*
DMR10	4	4858598	4864902	$1{,}49x10^{-8}$	54	*MSX1*
DMR11	6	133561368	133564578	$7{,}69x10^{-8}$	51	*EYA4*
DMR12	6	33156164	33178597	$5{,}97x10^{-7}$	251	*RNY4P10*
DMR13	13	28491326	28499045	$2{,}22x10^{-8}$	45	*PDX1*
DMR14	6	30649909	30659643	$6{,}13x10^{-7}$	104	*PPP1R18*
DMR15	1	10730684	10735169	$4{,}74x10^{-13}$	19	*CASZ1*
DMR16	16	51183363	51190201	$4{,}98x10^{-8}$	47	*AC009166.5*
DMR17	21	38076709	38083586	$4{,}19x10^{-10}$	26	*SIM2*
DMR18	10	118030292	118034357	$2{,}97x10^{-8}$	31	*GFRA1*
DMR19	2	63276183	63286621	$2{,}26x10^{-9}$	80	*OTX1*
DMR20	13	28500882	28503508	$3{,}09x10^{-13}$	14	*NA*

Abbreviations: AC009166.5: AC004866 gene (RNA Gene); CASZ1: Castor Zinc Finger 1; COL11A2: Collagen Type XI Alpha 2 Chain; EYA4: EYA Transcriptional Coactivator And Phosphatase 4; FDR: False discovery rate; GFRA1: GDNF Family Receptor Alpha 1; HOXA-AS2: HOXA Cluster Antisense RNA 2; MSX1: Msh Homeobox 1; NA: not available; OR2I1P: Olfactory Receptor Family 2 Subfamily I Member 1 Pseudogene; OTX1: Orthodenticle Homeobox 1; RNF5: Ring Finger Protein 5; RNF219-AS1: OBI1 Antisense RNA 1; PDX1: ; pancreatic and duodenal homeobox 1 PPP1R18: Protein Phosphatase 1 Regulatory Subunit 18; PTPRG-AS1: PTPRG Antisense RNA 1; RNY4P10: Pseudogene, RNY4 Pseudogene 10; SIM2: SIM BHLH Transcription Factor 2; TBX15: T-Box Transcription Factor 15; TNXB: tenascin XB protein;
